# Dual-Branch Multi-Dimensional Attention Mechanism for Joint Facial Expression Detection and Classification

**DOI:** 10.3390/s25123815

**Published:** 2025-06-18

**Authors:** Cheng Peng, Bohao Li, Kun Zou, Bowen Zhang, Genan Dai, Ah Chung Tsoi

**Affiliations:** 1 School of Computing, Zhongshan Institute, University of Electronic Science and Technology of China, Zhongshan 528402, China; cszoukun@zsc.edu.cn; 2 School of Computer Science and Engineering, University of Electronic Science and Technology of China, Chengdu 610000, China; lbh17@std.uestc.edu.cn; 3 College of Big Data and Internet, Shenzhen Technology University, Shenzhen 518118, China; zhangbowen@sztu.edu.cn (B.Z.); daigenan@sztu.edu.cn (G.D.); 4 School of Computing and Information Technology, University of Wollongong, Wollongong, NSW 2522, Australia; act@uow.edu.au

**Keywords:** deep learning, facial expression recognition, batch attention, attention fusion

## Abstract

This paper addresses the central issue arising from the (SDAC) of facial expressions, namely, to balance the competing demands of good global features for detection, and fine features for good facial expression classifications by replacing the feature extraction part of the “neck” network in the feature pyramid network in the You Only Look Once X (YOLOX) framework with a novel architecture involving three attention mechanisms—batch, channel, and neighborhood—which respectively explores the three input dimensions—batch, channel, and spatial. Correlations across a batch of images in the individual path of the dual incoming paths are first extracted by a self attention mechanism in the batch dimension; these two paths are fused together to consolidate their information and then split again into two separate paths; the information along the channel dimension is extracted using a generalized form of channel attention, an adaptive graph channel attention, which provides each element of the incoming signal with a weight that is adapted to the incoming signal. The combination of these two paths, together with two skip connections from the input to the batch attention to the output of the adaptive channel attention, then passes into a residual network, with neighborhood attention to extract fine features in the spatial dimension. This novel dual path architecture has been shown experimentally to achieve a better balance between the competing demands in an SDAC problem than other competing approaches. Ablation studies enable the determination of the relative importance of these three attention mechanisms. Competitive results are obtained on two non-aligned face expression recognition datasets, RAF-DB and SFEW, when compared with other state-of-the-art methods.

## 1. Introduction

Emotions are part and parcel of human existence. Many life decisions are made in various emotional states. Our inner emotional state manifests itself through both internal physiological signals and external behavioral expressions. Physiological signals include electrocardiac activity, pulse rate, and skin conductance, reflecting the body’s autonomic responses. Behavioral expressions encompass body gestures, vocal characteristics (prosody, tone), and crucially, facial expressions. Among these channels, facial expression is widely regarded as one of the most direct and revealing indicators of underlying emotion; it is said that as much as 55% of human communication is mediated through facial expressions [1]. Therefore, it is appropriate for Facial Expression Recognition (FER) to play a dominant role in the monitoring of human emotions.

With the emotional state being so integrated with the individual being, potential applications of emotion classification relate to daily living [1]. Typical application areas could be classified into the following: human–computer interface, enabling better communication between an intelligent devices and humans (see, e.g., [2] in the area of social robots); health monitoring, whereby the mental well being of the person could be monitored (see, e.g., [3] in austism screening); commercial applications (see, e.g., [4] for customer emotion recognition); well being monitoring (see, e.g., [5] in driver fatigue detection); computer mediated entertainment through computer games (see, e.g., [6,7] for computer game experience enhancement through facial expression recognition).

However, despite years of research and many promising practical application possibilities, automatic facial expression recognition remains largely an academic endeavor, rather than being included widespread practical applications. There are a number of reasons why the state of research and development of FER remains in its infancy: First, there are no universally accepted classifications of emotions [1,8]; there are the basic emotion models [9,10,11], the appraisal model [12,13], psychological construction model [14], and social construction model [15]. Secondly, the accurate classification of facial expression would require multiple non-invasive physiological measurements, which is often not available [16]. Thirdly, accurate determination of FER would require the existence of large labeled datasets (see, e.g., [17]), which is currently unavailable. Fourthly, emotion can change in a split moment; this makes the tracking of emotion in real time challenging (see, e.g., [10]).

In view of the many challenges inherent in Facial Expression Recognition (FER), researchers typically begin their investigations under the most stringent assumptions. Only after establishing solid baselines under these controlled conditions do they proceed to gradually relax these assumptions. Under such strict settings, datasets usually consist of center-aligned, cropped, single-face images captured under adequate lighting conditions. Furthermore, selected facial images are carefully chosen to manifest the underlying emotional expression clearly and unambiguously. A typical example of such a dataset is the CK (Cohn–Kanade) or its extended version, the CK+ dataset [18]. These datasets are derived from video recordings of individuals exhibiting expressions either voluntarily or involuntarily in response to visual stimuli. A suitable frame is manually selected from the video, center-aligned, normalized to a standard resolution, and cropped to minimize background interference. Multiple experts then annotate the images to reduce human labeling errors.

Using such datasets, classification accuracies are typically very high. In fact, some methods can even achieve near-perfect performance—up to 100% accuracy on test data—even under slightly relaxed conditions, such as selecting only the last frame from the video rather than “cherry-picking” the optimal one [19].

In the second tier of research, some assumptions are moderately relaxed. For example, lighting conditions might be suboptimal, and facial expressions may not be as exaggerated. However, the core assumption of having center-aligned and cropped faces usually remains. These cropped facial images are typically obtained through a two-step process: First detecting the face in the image, then normalizing and cropping it to a standard resolution. This preprocessing helps to reduce background noise and improve classification performance. Many FER datasets fall under this category (see [17] for a comprehensive overview), and most recent FER methods are evaluated using these datasets [19]. Notably, two commonly used datasets in this category are AffectNet [20] and the aligned version of RAF-DB [21]. However, classification accuracies drop significantly under these conditions—down to approximately 90% for RAF-DB and around 65% for AffectNet [19].

In the third and most challenging tier, even the assumption of center-aligned cropped faces is removed. Here, multiple faces may appear in a single image, with variations in pose, orientation, and scale. In such cases, simultaneous face detection and expression classification are required. This complicates FER significantly as most existing methods assume a single, center-cropped face. Evaluating performance in this setting requires metrics for both detection and classification accuracy.

In related fields such as object detection, there has been extensive research on simultaneous detection and classification using machine learning. A prime example is the YOLO (You Only Look Once) family of methods, designed for rapid object detection and classification (e.g., [22,23,24,25,26,27,28]). Earlier YOLO versions (e.g., [22,23]) lacked a dedicated feature enhancement mechanism in the neck network to refine features extracted by the backbone.

However, few studies have applied YOLO-based methodologies to FER [29,30], possibly due to the complexity of capturing minute muscle movements across various facial regions—features essential for accurate expression recognition. Unlike general object detection, FER demands both the identification of the face and the precise analysis of localized muscle movements. Therefore, YOLO architectures used in FER must be capable of handling multi-scale features effectively. This requirement is addressed by the YOLOX architecture [25], which is employed in the few existing FER studies focused on Simultaneous Detection and Classification (SDAC) [29,30].

The YOLOX architecture follows the same design philosophy as its predecessors and consists of three components: The backbone network, which generates feature maps from the input image; the neck network, which further processes these features; and the head network, which outputs the classification results, bounding box regressions, and objectness scores. Performance is typically evaluated using mean Average Precision (mAP).

The primary design challenge in applying YOLOX to FER lies in the neck network. The neck must balance two competing demands: (1) extracting robust global features to support accurate object detection and (2) preserving fine-grained local features necessary to distinguish subtle facial expressions. Achieving this balance is non-trivial.

The neck network in YOLOX is tasked with extracting suitable features at each scale from the multi-scale signals produced by the backbone. A naïve solution would be to use only the features from a specific scale. However, this neglects valuable cross-scale information. A more effective approach involves integrating information from adjacent scales through upsampling or downsampling so that features from neighboring resolutions can be aligned and fused. Typically, two input signals are used—one at the target scale and another rescaled from a neighboring level. These are pre-processed and passed through a feature extractor that outputs refined features for that scale, which are then fed into the head network.

This paper focuses on the design of that feature extractor. In summary, the effectiveness of simultaneous detection and classification in FER hinges critically on the quality of features extracted in the neck network. Therefore, the core contribution of this work lies in the development of an effective multi-scale feature extraction mechanism tailored for FER within the YOLOX framework.

In this paper, we introduce a novel feature extraction model that takes into account three aspects of the incoming signal, which is BS×C×H×W, where *BS* denotes the batch size, *C* the number of channels, and H×W, the spatial dimension: Batch Attention (BA) [31,32], which extracts features along the batch dimension; Channel Attention (CA) [33], which extracts features in the channel dimension; and Neighborhood Attention (NA) [34], which extracts features in the spatial dimension. The features extracted by BA [31,32] exploit the information available in a batch of incoming images and how these relate to one another across images in the batch. The CA we used provides an individual weight for each element in the BS×C×H×W, and these weights change epoch by epoch, thereby allowing the features extracted in the channel dimension to adapt to the incoming images epoch by epoch. This is called Adaptive Graph Channel Attention (AGCA) [33]; as in the modeling, it adopts a graph formulation in providing the connectivity of one channel with that of other channels. AGCA is a significant advancement from the usual channel attention mechanisms, like the Squeeze and Excitation (SE) method [35], which provides an overall weight for each channel, and the Efficient Channel Attention (ECA) method [36], which provides an adaptive convolutional kernel in the channel dimension. The NA [34] works on the spatial dimension using a local neighborhood that can be thought of as Self Attention (SA), except that it works in a local neighborhood instead of across the entire H×W dimension. AGCA is different to the other previously published attempts in deploying the YOLOX framework for FER [29,30], none of which consider exploiting the information available in a batch of images, while in practice the processing is normally conducted in a batch. The AGCA method is a significant improvement on other CA methods. The NA replaces the awkward assumption in FER-NCA [30], where it is assumed that the facial features are in vertical and horizontal dimensions, in the x-y coordinate dimensions. This makes it awkward when the facial sites are not aligned on the same horizontal and vertical axes, and this occurs frequently in real-life situations.

We have applied this proposed method to two un-aligned FER datasets, RAF-DB [21] and SFEW [37], and found that this produces State-of-the-Art (SOTA) results when compared with other models [29,30] using the same YOLOX framework for the FER of un-aligned facial images. The contributions of this paper may be expressed as follows:

- Novel Application of Attention Mechanisms: While batch attention, AGCA, and NA are not novel inventions, this work represents their first deployment in simultaneous facial expression detection and classification. Specifically, we introduce (i) batch attention for this dual task, (ii) AGCA with adaptive channel weighting for FER, and (iii) NA integrated into residual networks for spatial processing in FER.
- First Integrated Architecture: The design integrating these three attention mechanisms within a dual-input stream framework, processing features through YOLOX’s head network, constitutes the first such architecture across any application domain. This model achieves state-of-the-art results on challenging non-aligned FER datasets (RAF-DB and SFEW).
- Proven Generalization Capability: The model demonstrates strong generalization to real-world scenarios, validated on unseen "in-the-wild" test data not encountered during training, confirming its practical robustness beyond benchmark datasets.

The rest of this paper is organized as follows: In Section 2, some related work on our proposed model is given. Section 3 gives an exposition of the methodology employed in the design of our proposed model. Section 4 provides information on the datasets we used, the experimental setup, visualization of the results obtained by applying our proposed model on two real-life datasets, its performance when compared with other state-of-the-art methods, comparison of the misclassification results and misdetection results on applying both our proposed model and the FER-NCAMamba model on the same dataset, RAF-DB, and finally a discussion on the limitations of our proposed model. Section 5 draws some conclusions on our proposed model and gives some future directions of research on this topic.

## 2. Related Work

In this section, we will provide a brief overview of some work related to this study. Section 2.1 will introduce the relevant work on Facial Expression Recognition (FER) [38]. The YOLO framework [39] will be briefly discussed in Section 2.2. Next, Section 2.3 will cover topics related to attention mechanisms [40]. Finally, Section 2.4 will address issues related to FER datasets.

### 2.1. Facial Expression Recognition

In recent years, significant progress has been made in the area of Facial Expression Recognition (FER) [38,41,42]. All of these models followed the classic recognition model, which consists of a feature extraction part, followed by a classifier. These developments are driven by combined advancements in traditional methods and deep learning approaches. Traditional FER methods primarily rely on handcrafted features and one or two fully connected layers as a classifier. There are a number of possible handcrafted features: Spatial features, e.g., Histogram of Oriented Gradients (HOG) [43], Histogram of Oriented Optical Flow (HOF) [44], features related to texture (see, e.g., [45]). For example, regarding the topic of texture features, a series of developments, commencing with: Zhao et al. [46], extended the Local Binary Pattern (LBP) [45], a method that is used for static texture analysis, to the spatio-temporal domain, e.g., extracting texture features in videos, by proposing the volume local binary pattern for dynamic texture analysis. Ahmed et al. [47] introduced the Directional Ternary Pattern (DTP) encoding method, which computes the edge response of the central pixel in various directions and generates a ternary code for each neighboring pixel, effectively encoding local texture while preserving more information. Building on DTP, Ryu et al. [48] proposed the Local Directional Ternary Pattern (LDTP), which combines directional information with ternary patterns to provide more stable edge features in edge regions and overcome the limitations of edge-based methods in smooth areas.

Deep learning approaches, particularly Convolutional Neural Networks (CNNs), have become the dominant method in FER, owing to their success in image recognition tasks. Unlike traditional methods, CNNs automatically learn deep feature representations, while the classifier used is often composed of simple multiple fully connected feedforward layers, significantly enhancing the recognition accuracy [19]. It has been found that, in one particular type of deep learning network, the Visual Geometry Group (VGG) of the University of Oxford [49], the first few layers emulate the classical handcrafted spatial features, e.g, HOG, HOF, thus providing good confidence that such deep learning networks could outperform those that are dependent on handcrafted features. There are many deep learning networks. For example, two popular deep learning models were customized: a method based on the VGG network [50] was customized for FER, and a method based on ResNet (Residual Network) [51] was again customized for FER, delivering strong performance on datasets like FER-2013 and CK+. However, these models do not focus on the possibility of multiple regions on the face to compose an expression, and therefore their accuracies in classifications would be rather limited. Some researchers use hybrid frameworks to boost precision [52]; others draw on contrastive region relevance learning [53], leveraging contrastive learning to highlight key facial region differences and improve expression variation recognition.

To overcome these limitations, researchers have explored multi-level feature fusion techniques to improve feature representation capabilities, see, e.g., [54], which, apart from extracting features over multiple scales, also integrate features from various network layers. Such methods capture both local facial details and global structures, thus improving the model when compared with those that only extract features at the same scale. Other examples of extracting multi-scale features include Lin et al. [55], who proposed a feature pyramid structure that uses multi-scale feature fusion to enhance the recognition of subtle expressions. Facial landmark detection [54] results can help increase the FER accuracy.

Recently, the self-attention mechanism [56], commonly referred to as the transformer model, has gained significant traction in tasks such as image classification and natural language processing. These techniques have also been applied to FER, helping models focus more effectively on critical facial regions. For example, in the area of dynamic FER, Huang et al. [57] captured key points in the spatial region and self attention in the temporal dimension and fused them so as to extract features in both the spatial and temporal dimensions.

### 2.2. YOLO

YOLO (You Only Look Once) [39] is an efficient real-time object detection algorithm first proposed by Redmon and Farhadi [58], marking a significant breakthrough in the areas of object detection and classification. This has become a standard framework for simultaneous detection and classification tasks. Unlike traditional two-stage detection methods (e.g., the R-CNN series [59]), YOLO [39] reformulates object detection as a single-stage regression problem, directly predicting the positions and classes of objects from the entire image. This approach eliminates the need for complex region proposal generation, thus improving detection speed and enabling good performance in real-time scenarios.

The core idea of YOLO [39] is to divide the input image into a grid, with each grid cell responsible for predicting the bounding boxes and class probabilities of objects within it. Optimizing the entire image, YOLO [39] captures global contextual information between objects and their background, effectively reducing false positives. Moreover, YOLO [39] performs object classification and localization simultaneously in a single forward pass, resulting in high inference speed, which makes it particularly suitable for latency-sensitive applications.

Through multiple iterations, the YOLO family has achieved a balance between detection accuracy and speed. For instance, YOLOv2 [58] introduced anchor boxes and multi-class detection capabilities; YOLOv3 [22] leveraged a Feature Pyramid Network (FPN) [55] structure to enhance the detection of small objects through the use of a multi-scale framework; YOLOv4 [23] incorporated techniques such as Mosaic data augmentation and CSPNet (Cross-Stage Partial Network) [60], a variant of ResNet (Residual Network), further optimizing detection performance; and YOLOv5 gained popularity for its ease of use and deployment-friendly nature. These advancements enable the YOLO family to deliver robust detection capabilities across diverse scenarios.

However, despite all its improvements on detection capabilities, it appears that not much attention has been paid to the classification aspect of the simultaneous detection and classification problem in using the YOLO framework, in particular, the feature extraction aspect in the neck network.

### 2.3. Attention Mechanisms

In recent years, attention mechanisms [19,40] have significantly advanced the field of computer vision, playing a crucial role in numerous computer vision tasks. Initially introduced to emulate the human visual system’s ability to identify salient regions, attention mechanisms dynamically adjust weights based on input image features. This has led to remarkable achievements in tasks such as image classification, object detection, image generation, and video understanding.

The attention mechanism [40] is a core deep learning technique designed to dynamically allocate weights to features, enhancing the model’s focus on critical information. There are many attention mechanisms that have been proposed [40]. There are three particular attention mechanisms that are of interest to this paper: (1) Batch Attention (BA) [31] calculates the correlation between different samples within a batch, capturing global information across samples. This is particularly beneficial for tasks involving group behavior modeling and inter-sample dependencies. Neighborhood Attention (NA) [34] focuses on the local region features by limiting the self-attention computation to a neighborhood, effectively capturing local details while reducing computational overhead. This is particularly suitable for efficient vision tasks and scenarios like facial expression recognition, where handling local variations is essential. Channel Attention, in particular Adaptive Graph Channel Attention (AGCA) [33], dynamically adapt each weight to an input (*BS*, *C*, *H*, *W*), where *BS* is the batch size, *C* is the number of channels, and *H* and *W* are, respectively, the height and width in the spatial dimension. BA and NA may be considered as an extension of the self attention introduced in [56] while AGCA may be considered an extension of the “squeeze and excitation” method of channel attention [35].

### 2.4. Facial Expression Datasets

There are numerous distinctive datasets in the area of facial expression recognition, such as AFEW, SFEW, MELD, AffWild, and RAF-DB [61]. These datasets vary in scale and characteristics, with the aim of comprehensively capturing facial expressions as they occur in real-world scenarios. Various research methods have been proposed to analyze these datasets. For further information on these datasets and related studies, refer to the relevant literature [61].

Moreover, to evaluate the generalizability of the trained models to a real-life scenario, the trained model could be applied to images that are not collected in these two datasets: RAF-DB and SFEW. To that end, we have collected a few samples of people in the wild. This additional real-life dataset would be used in the evaluation of generalizability on unseen data.

In this paper, we focus on two “real-world facial expression” datasets, namely RAF-DB [21] and SFEW [37]. Furthermore, we utilize some of the most recent advanced deep learning methods, such as BA [31], NA [34], and CA [33].

## 3. Method

The organization of this section is as follows: In Section 3.1, an overview of the methodology used in this paper will be provided. This is then followed by a brief overview of the main components of the YOLOX framework. This is followed by a discussion of the components in the design of the feature extractor in Section 3.3, leading to a description of the overall design of the feature extractor where the components are assembled to give an architecture of the feature extractor in Section 3.4.

### 3.1. Overview

**Figure 1:**
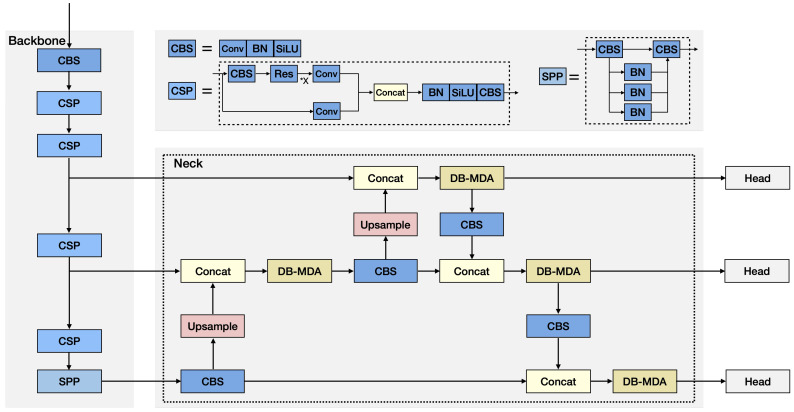
Illustration of the overall architecture. The DB-MDA module refers to the Dual-Branch Multidimensional Attention unit. “Concat” is a convenient way to depict the two separate incoming signals in this diagram. It does not imply they are combined at this point. The term Head corresponds to the head structure in YOLOX, where each head independently handles classification, detection, and regression tasks [25]. The detail of this is shown in Figure 2.

In this paper, we focus on the joint learning task of the Simultaneous Detection and Classification (SDAC) of facial expressions. First, we adopt the YOLOX framework [25], as shown in Figure 1, which consists of three sections: the multi-scale signal generation section (“backbone” network), the feature extraction section (“neck” network), and the classification and detection section (“head” network). The backbone network is essentially responsible for generating three signals: coarse scale, medium scale, and fine scale signals from a given input image. The neck network extracts features from these three signals of different scale, one feature extractor for each scale, and then outputs the features extracted on three scales to the head network. The head network applies detection and classification to each of these three sets of features extracted and provides the Region of Interest (ROI) in terms of bounding boxes and likely class of objects contained in the ROI. The final detected ROIs are selected from these detected ROIs and the classification of objects within each selected ROI. The unknown weights introduced in the model are trained on a loss function consisting of the following: (1) the classification (cls) category prediction uses cross-entropy loss; (2) the regression (reg) for bounding box localization uses IoU (Intersection over Union) loss, a measure of the overlap between the bounding box and the ground truth information on the object; (3) the objectness (obj) confidence, indicating the probability of object presence, also uses cross-entropy loss.

Our design work focuses mainly on the feature extraction module in the neck network, while the multi-scale signal generation module, the backbone network, and the head network will only be briefly mentioned as these two networks are the same as the ones in [25].

### 3.2. The YOLOX Framework

The YOLOX framework is shown in Figure 1, and each section is described in the following subsections.

#### 3.2.1. Backbone Network

The backbone network may be described as follows:

(1) $$\left(S_{\ell },S_{m},S_{h}\right)=F_{b}\left(I\right)$$

where I is the input image; $S_{\ell }$, $S_{m}$$S_{h}$ are, respectively, the coarse scale, medium scale, and fine scale output signals from the backbone network; and $F_{b}\left(\cdot \right)$ denotes the backbone network. The details of $F_{b}\left(\cdot \right)$ can be found in [25].

#### 3.2.2. Neck Network

The neck network may be described by the following equations:

(2) $$\begin{array}{ccc} x_{1} & = & F(S_{\ell },x_{2}\left(↓\right)) \end{array}$$

(3) $$\begin{array}{ccc} z & = & F(S_{m},S_{\ell }\left(↑\right)) \end{array}$$

(4) $$\begin{array}{ccc} x_{2} & = & F(z,x_{3}\left(↓\right)) \end{array}$$

(5) $$\begin{array}{ccc} x_{3} & = & F(S_{h},z\left(↑\right)) \end{array}$$

where $x_{1}$, $x_{2}$, and $x_{3}$ are, respectively, the outputs of the neck network and z is the intermediate signal generated in the medium scale branch. This could have been subsumed in the equation for $x_{2}$, but we chose to make it explicit to clearly show the signal flow. $x_{2}\left(↓\right)$ and $x_{3}\left(↓\right)$ are, respectively, the down sampled versions of $x_{2}$ and $x_{3}$. $S_{\ell }\left(↑\right)$ and z(↑) are, respectively, the upsampled versions of $S_{\ell }$ and z. Note that here, to make the relationships clear, we have ignored the representation of the CBS components. F(·,·) clearly depicts the two incoming signals. It should be noted that the architecture of these dual incoming paths, F(·,·), is the same for all paths in the neck network. The design of F(·,·) will be discussed in more detail in later subsections of this section, as this is the main innovation in this paper.

#### 3.2.3. Head Network

**Figure 2:**
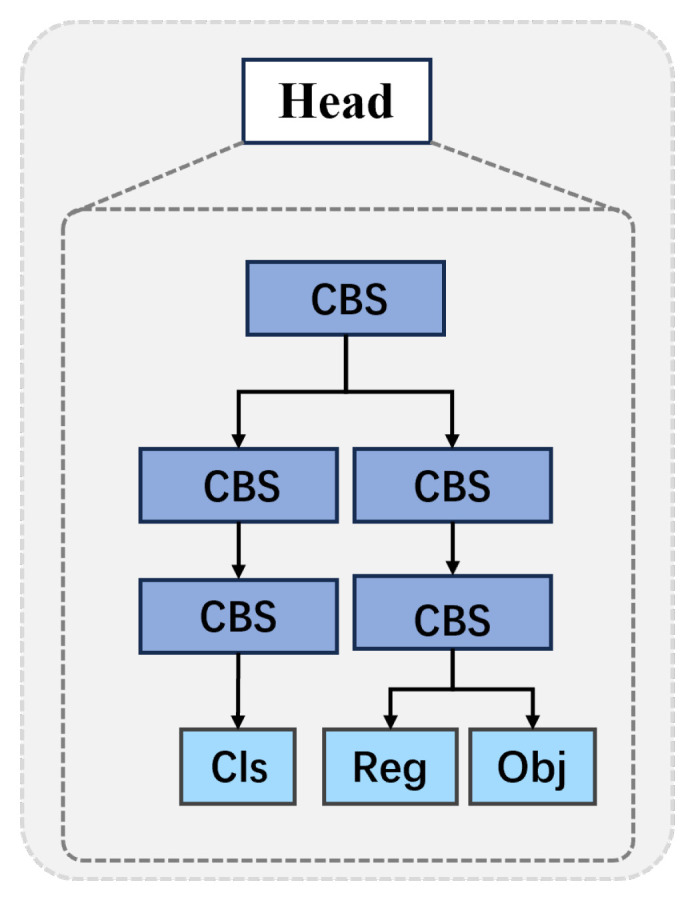
The structure of an individual head. CBS stands for the connection of three components: convolutional layer, batch normalization, and SiLU (Sigmoid Linear Unit), as shown in Figure 2.

Figure 2 shows the structure of an individual head in the head network.

There are three heads, as follows:

(6) $$\begin{array}{ccc} \left(cls_{\ell },reg_{\ell },obj_{\ell }\right) & = & H(x_{1}) \end{array}$$

(7) $$\begin{array}{ccc} \left(cls_{m},reg_{m},obj_{m}\right) & = & H(x_{2}) \end{array}$$

(8) $$\begin{array}{ccc} \left(cls_{h},reg_{h},obj_{h}\right) & = & H(x_{3}) \end{array}$$

where $cls_{k}$, $reg_{k}$, $obj_{k}$, and k=ℓ,m,h are, respectively, the predicted class of the object within the bounding box, the regression for bounding box location, and the objective confidence of the coarse scale, medium scale, and fine scale, respectively. The details of H(·) can be found in [25]. If obj>τ, where τ is a preset threshold, then the corresponding reg bounding box, together with the predicted classification of the object within the bounding box, will be the output of the model, i.e., the output of the SDAC for the given input image I. Note that, because the threshold τ is a preset constant, in a given image there could be more than one bounding boxes could satisfy the condition; where more than one bounding box satisfies this condition, there is no guarantee that the different bounding boxes are nested, i.e., the multiple bounding boxes are nonoverlapping. This will be flagged as an error, as for a human face it is anatomically impossible for the same face to occur in two or more nonoverlapping locations within the same scale in the image.

### 3.3. Structure of the Feature Extractor

As is clear from Figure 1, the feature extractor $F(in_{1},in_{2})$, where $in_{1}$ and $in_{2}$ are two incoming streams, on the same scale, is the key characteristic of our model.

**Figure 3:**
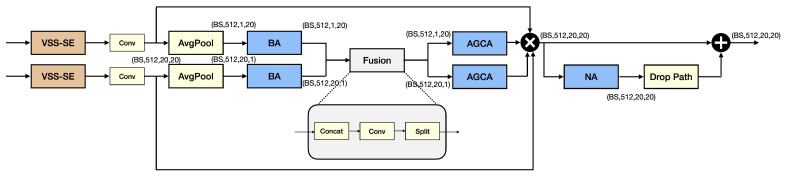
The overall structure of the proposed Dual-Branch Multidimensional Attention (DB-MDA) feature extractor, where VSS-SE denotes the pre-conditioning module, BA denotes the batch attention, AGCA denotes Adaptive Graph Channel Attention, and NA denotes the Neighborhood Attention; Drop Path denotes the drop path strategy, which serves the role of a regularizer in the training of the unknown weights in the extractor.

Figure 3 shows the overall structure of this feature extractor $F(in_{1},in_{2})$. This structure may be broken down into the following modules: pre-conditioning module, denoted as VSS−SE+Conv, where VSS is short for Visual State Space and SE is short for Squeeze and Excitation, in Figure 3; the BA-AGCA (Batch Attention–Adaptive Graph Channel Attention) module. This is followed by a residual network with NA (Neighborhood Attention) serving in the residual block. To emphasize the importance of the two input signal characteristics of this architecture, we will call this a Dual Branch–Multidimensional Attention (DB-MDA) module with two submodules: the preconditioning unit, consisting of the VSS-SE unit, and the attention mechanism unit, consisting of the BA-AGCA unit followed by the NA residual network.

#### 3.3.1. Pre-Conditioning Unit: Parallel Structure of VSS and SE

**Figure 4:**
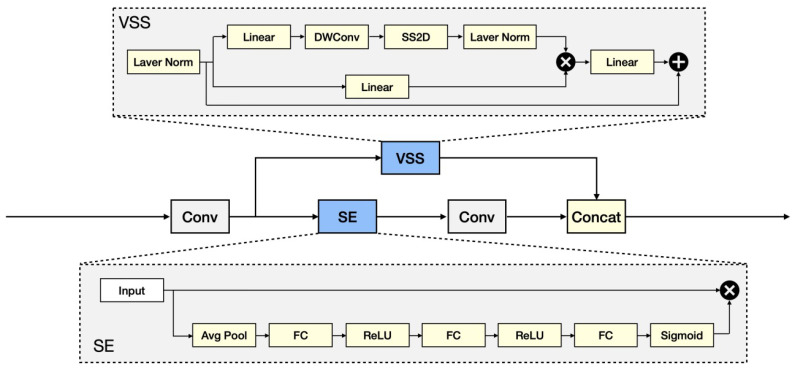
Illustration of the parallel structure of VSS and SE.

Figure 4 shows the details of the pre-conditioning unit. In Figure 4, the VSS module and the SE module are crucial components of our enhancement section, responsible for global information modeling and inter-channel relationship modeling, respectively. The detailed design of this unit follows that in [30].

VSS module

The VSS module is inspired by the state-space model [62,63] and aims to dynamically model the input visual features. It effectively captures global contextual information through a state update mechanism, while keeping the computational complexity linearly increasing. This efficient design allows the VSS module to expand the receptive field when processing high-dimensional data, providing more comprehensive global feature representations for the network, which is particularly suitable for handling subtle facial expression changes and complex scenes. Inspired by S6 (Selective Scanning Mechanism), the VSS module incorporates a mechanism called 2D Selective Scanning (SS2D). This mechanism generates dynamic weights from the input information, enhancing flexibility and adaptability. Additionally, to address directional sensitivity, the VSS module introduces a Cross Scanning Module (CSM), which scans the input image features in four different directions, transforms these features into sequences, and integrates them. This ensures that each pixel absorbs contextual information from multiple directions, enhancing the model’s ability to capture global information.

SE Module

The SE module focuses on modeling the importance of inter-channel relationships. It achieves this in two core steps: first, it compresses the spatial information of the features through global average pooling (Squeeze), and then dynamically generates channel weights using a fully connected layer and activation function (Excitation). These weights are used to adjust the contribution of each channel, allowing the model to highlight key features while suppressing redundant information.

The specific details of the module are as follows:

Assume the shape of the input feature map is $X\in R^{C\times H\times W}$, where C is the number of channels and H and W are the height and width, respectively. Compress spatial dimensions through Global Average Pooling (GAP) to generate channel descriptors $z\in R^{C\times 1\times 1}$.

(9) $$z_{c}=\frac{1}{H\times W}\sum_{i=1}^{H}\sum_{j=1}^{W}X_{c}\left(i,j\right)$$

where c=1,2,…,C.

Generate channel weights $s\in R^{C\times 1\times 1}$ using a Fully Connected (FC) layer and non-linear activation functions (such as ReLU and sigmoid).

(10) $$s=sigmoid(W_{2}ReLU\left(W_{1}z\right))$$

where sigmoid(·) is the sigmoid function and $W_{1}$ and $W_{2}$ are training weights.

Multiply the channel weights s with the input feature map X to obtain the output $X_{\mathrm{SE}}\in R^{C\times H\times W}$ of the SE branch.

(11) $$X_{SE,i}=s_{i}X_{i}$$

where i=1,2,…,C and $X_{SE,i}$, $X_{i}$ represent, respectively, the *i*-th channel of H×W. In other words, each channel is multiplied by a weight $s_{i}$ obtained from Equation (10).

The output $X_{SE}$ of the SE branch and the output $X_{VSS}$ from the VSS branch are weighted and fused to obtain the final output feature map $X_{out}$.

(12) $$X_{out}=\alpha X_{SE}+\beta X_{VSS}$$

where α and β are the learnable weights used to balance the contributions of the two branches.

To succinctly represent this pre-conditioning unit, we have:

(13) $$X_{out}=F_{VSS//SE}\left(X_{in}\right)$$

where $F_{VSS//SE}\left(\cdot \right)$ denotes the complex action involving either branch to capture global information and to assign relative importance to the channels.

#### 3.3.2. Attention Mechanism

Here we group the description of each individual attention mechanism used in this paper. As BA (Batch Attention) and NA (Neighborhood Attention) are extensions of SA (Self Attention), we therefore describe BA and NA first before describing AGCA (Adaptive Graph Channel Attention).

Self Attention [56]

The input signal is $x\in R^{BS\times C\times H\times W}$, representing a batch of *BS* images, each with *C* channels and spatial dimensions H×W. The spatial dimensions are first flattened into vectors of length *HW*, transforming each image into $X\in R^{HW\times C}$. This is then embedded using projection matrices $W_{Q}\in R^{C\times d}$, $W_{K}\in R^{C\times d}$, and $W_{V}\in R^{C\times d}$ to obtain $Q=XW_{Q}\in R^{HW\times d},K=XW_{K}\in R^{HW\times d},V=XW_{V}\in R^{HW\times d}$. Self attention SA is calculated as:

(14) $$\mathrm{SA}=\sigma \left(\frac{QK^{T}}{\sqrt{d}}\right)V$$

where $\mathrm{SA}\in R^{HW\times d}$ and σ(·) denotes the softmax function applied row-wise. To reconstruct spatial dimensions, the HW×d matrix is transformed using two projection matrices, $W_{1}\in R^{d\times HW}$ and $W_{2}\in R^{HW\times HW}$:

(15) $$Y=\mathrm{SA}W_{1}W_{2}\;\mathrm{with}\;Y\in R^{HW\times HW}.$$

The output Y is then reshaped as $R^{C\times H\times W}$. Upon aggregation across the batch dimension *BS*, the final product retains the original dimensions BS×C×H×W.

This process can be viewed as holding the batch dimension *BS* constant while processing each C×H×W sample. The channel-wise flattened representation $X\in R^{C\times HW}$ is used to compute Q, K, and V, followed by the self-attention operation in Equation (14). The resulting HW×HW matrix SA is then reshaped to $R^{H\times W}$ per channel. The *BS* samples of C×H×W are stacked to form the output tensor BS×C×H×W. The entire operation is compactly represented as $Y=F_{SA}\left(X\right)$, where both X and Y are $\in R^{BS\times C\times H\times W}$, and $F_{SA}\left(\cdot \right)$ denotes the self-attention transformation.

Batch Attention [31,32]

This may be considered as forming self attention along the batch dimension. The process is very similar to the self attention formed along the channel dimension. The easiest way to see how this can be achieved is to swap the position of *BS* and *C* on the input $x\in R^{BS\times C\times H\times W}$ to become $x^{\prime }\in R^{C\times BS\times H\times W}$. Then again flatten H×W into a row vector 1×HW, and then we obtain a transformed input $X^{\prime }\in R^{HW\times BS}$. Then, it is possible to embed this input $X^{\prime }$ in three matrices, $Q=X^{\prime }W_{Q}$, $K=X^{\prime }W_{K}$, and $V=X^{\prime }W_{V}$; all W$\in R^{BS\times d}$ and Q, K, and V are $\in R^{HW\times d}$. Then, we may form SA using the formula (Equation (14)). Then, it is possible to pre-multiply by $W_{1}^{\prime }$ and post multiply by $W_{2}^{\prime }$ the SA matrix and transform it back into H×W for each sample in the batch. Then, the BS×H×W is assembled together for each channel to obtain C×BS×H×W. Finally, perform a transpose to obtain the output in the shape of BS×C×H×W.

This can be represented by $Y=F_{BA}\left(X\right)$, where $F_{BA}\left(\cdot \right)$ is a convenient way to summarize the procedure indicated here to obtain $Y\in R^{BS\times C\times H\times W}$ from the input $X\in R^{BS\times C\times H\times W}$.

Neighborhood Attention [34]

Consider a local neighborhood h×h. This can be flattened into a vector $1\times h^{2}$. So we have a Q-neighborhood $h^{2}\times d_{Q}$, K-neighborhood $h^{2}\times d_{K}$, and a V-neighborhood $h^{2}\times d_{V}$.

Then, it is possible to form the $h^{2}\times h^{2}$ matrix SA using the formula (see Equation (14)) of each Q-neighborhood, K-neighborhood, and V-neighborhood through the embedding process shown in the self-attention paragraph above. This will result in $d_{Q}d_{K}d_{V}=d^{3}$ SA’s, if $d_{Q}=d_{K}=d_{V}=d$. Each SA $\left(h^{2}\times h^{2}\right)$ is then converted back into H×W through pre-multiplication and post-multiplication, as shown in the above self-attention paragraph, and thus we will have C×H×W. Assemble all the samples that we will have BS×C×H×W.

So, we have $Y=F_{NA}\left(X\right)$, where Y and X are $\in R^{BS\times C\times H\times W}$, and $F_{NA}\left(\cdot \right)$ is a convenient way to summarize the procedures taken in the formation of the NA with a specified neighborhood h×h. The NA may be considered as a generalization of SA, as when h=H=W; the NA then collapses back into an SA.

Adaptive Graph Channel Attention [33]

Our channel attention mechanism is an adaptive channel attention in the style of Adaptive Graph Channel Attention (AGCA) proposed in [33].

Given an input C×H×W image, the AGCA may be considered as consisting of two parts: (1) the feature mapping layer, which consists of two linear embedding functions, $F_{1}$ and $F_{2}$, respectively, and (2) the determination of the weights associated with these feature vectors. The input first goes through a global average pooling operation that renders an output C×1×1 from the input C×H×W. These C×1×1 vectors then go through a feature mapping layer, $F_{1}\left(\cdot \right)$, which is a linear embedding function of the form $f\left(x_{out}\right)=Wx_{in}$, where $W\in R^{h\times C}$ and h<C. The AGCM module is a module that will consider these *h* feature vectors as the vertices of a graph. As nothing is known about this graph, it is simplest to consider it to be fully connected, i.e., each vertex of the graph is connected to every other vertex in the graph. As this is a graph, the input and output of the graph are related through an adjacency matrix. The connection weights among the fully connected graphs may be formed. The weights of each feature vertex are obtained through an Adaptive Graph Convolutional Module (AGCM). These weights are mapped back to the original feature map as channel weights, through another linear embedding $F_{2}\left(\cdot \right)$ from a vector of length *h* back into a vector of length *C*.

(16) $$y=x⊙sigmoid\left(F_{2}\left(ReLU\left(G\left(F_{1}\left(GAP\left(x\right)\right),A\right)\right)\right)\right)$$

where ⊙ denotes multiplication by element of the transmission. In other words, AGCA finds a weight for each element in the input C×H×W through Equation (16). The linear embedding functions $F_{1}\left(\cdot \right)$ maps the number of channels *C* to a lower dimension, *n*, i.e., $h=W_{1}x$, and $x\in R^{C}$, $h\in R^{h}$, and $F_{2}\left(\cdot \right)$ is doing the expansion from *n* vertices back into *C* vertices, provide what is known as a bottleneck structure and the AGCM is the “sandwich” between these two linear embedding functions. The graph formed by the *n* vertices is completely connected, i.e., each vertex is connected to every other vertex, and the output is formed by all these *n* vertices connecting to the *C* vertices. So if we ignore the ReLU, Equation (16) may be conceptualized as the *C* vertices being projected to *n* vertices (by the linear embedding $F_{1}\left(\cdot \right)$); the graph formed by these *n* vertices is complete, i.e., each vertex is connected to every other vertex in the graph, and then the *n* vertices are mapped back onto *C* vertices through the linear embedding mapping $F_{2}\left(\cdot \right)$. Since the graph of *n* vertices is complete, it is possible to express the outputs of this graph by an adjacent matrix A, operating on the *n* vertices.

For AGCM, it is useful to conceptualize the output of the first linear embedding $F_{1}\left(GAP\left(x\right)\right)$ as a graph consisting of *n* vertices, where each vertex represents a channel where n<C. The edge $e_{ij}$ is a connection between the vertex *i* and vertex *j*. This edge has a weight $w_{ij}$ associated with it. If we assume that each vertex $v_{i}$, i=1,2,…,n, of the graph, there is a feature vector $f\left(v_{i}\right)\in R^{m\times 1}$. Then, for a vertex $v_{i}$, with a connection configuration $B_{i}$, which collects all the incoming edges from other vertices, $v_{j}$, then we will have:

(17) $$f\left(v_{i}\right)=\sum_{v_{j}\in B_{i}}w_{ij}f\left(v_{j}\right)$$

for i=1,2,…,n. The n×n matrix A formed is known as the adjacency matrix. This matrix denotes the connectivity among the *n* vertices. For each vertex *i*, the number of incoming edges is known as the degree of vertex *i*. For the n×n matrix A, there exists an n×n diagonal matrix D known as the degree matrix, the diagonal elements of this diagonal matrix $d_{ii}=\sum_{j}A_{ij}$, where $A_{ij}$ is the i,j-th element of the adjacency matrix A. As it is possible that a particular *i* vertex has no incoming edge, so $D_{ii}$ would be 0. It is common to normalize the adjacency matrix A, which would involve the inverse of the degree matrix D. So to prevent division by a 0 degree vertex from happening, it is usual to add a small constant ϵ to each of the diagonal elements of D to prevent the situation when a vertex does not have any incoming edge.

Now as we have *n* vertices, and each vertex has an m×1 feature vector, then the m×n feature map of this graph is Z. Then, it is possible to express Equation (17) as follows:

(18) $$Z^{o}=WZ^{i}\tilde{A}$$

where $\tilde{A}=D^{\left(-\frac{1}{2}\right)}AD^{\left(-\frac{1}{2}\right)}$, A is the adjacency matrix, $Z^{i}$, m×n, and $Z^{o}$, m×n, are, respectively, the input and output feature maps. W is m×m; it is the weights of the 1D convolutional layers to represent the set of weights $w_{ij}$ in Equation (17) for each vertex.

**Figure 5:**
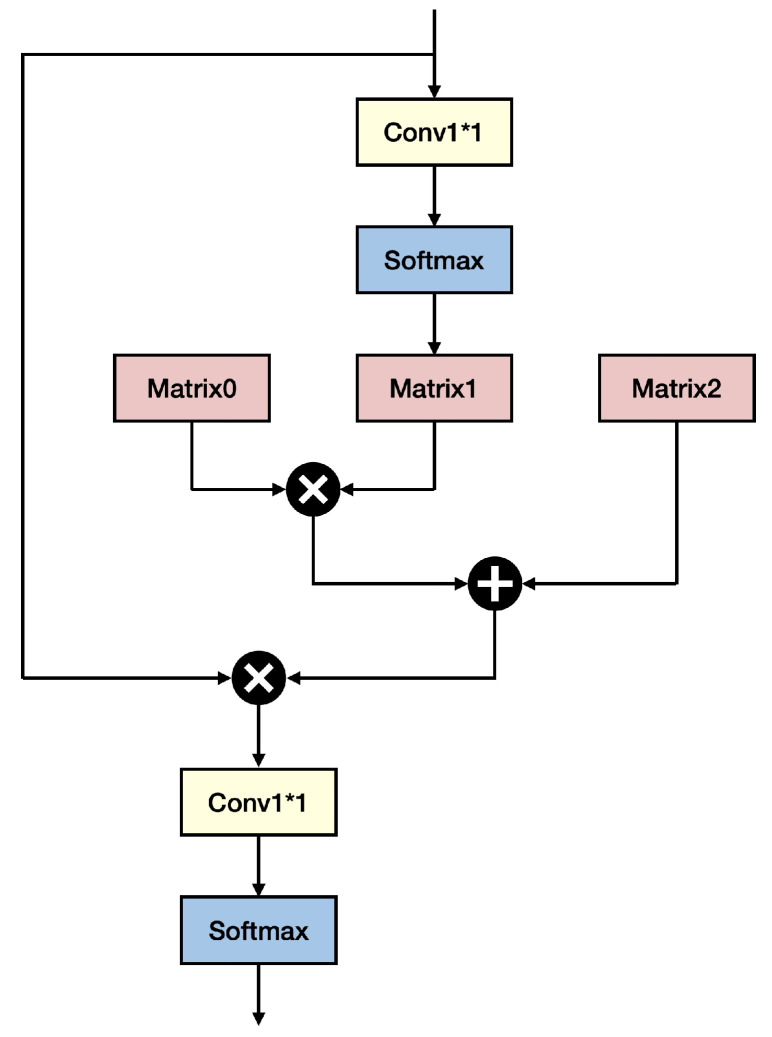
Illustration of the relationship of $\tilde{A}$ with $A_{0}$, $A_{1}$, and $A_{2}$. Please see main text for an explanation of these symbols, The asterisk denotes multiplication.

The normalized adjacency matrix $\tilde{A}$ can be decomposed into three matrices, $\tilde{A}=A_{0}\times A_{1}+A_{2}$, where $A_{0}=I$, $A_{1}$ is a diagonal matrix, and $A_{2}$ is a matrix without any specific structure (please see Figure 5 for an illustration of this relationship).

This decomposition is general in the sense that in an n×n matrix, R can always be decomposed into two matrices, one representing the diagonal elements D and the other representing the off diagonal elements, and it has all 0 diagonal elements Q, where $Q_{ii}=0$ and $Q_{ij}\neq 0$ for all *i* and *j*. In the current formulation, there is no way in which we could guarantee that the diagonal elements found would be 0, and so we conjectured that, by initializing the elements of the $A_{2}$ matrix to be very small, and then using backprop error to update the weights from one epoch to another, that through such action the diagonal elements as well as the off-diagonal elements of $A_{2}$ would not grow too rapidly. This conjecture is found to be valid in all the experiments we conducted in this paper. Put differently, if we initialize the $A_{2}$ as close to 0, i.e., $\tilde{A}$ is assumed to be diagonally dominant, then this diagonal dominancy would be maintained even when $\tilde{A}$ is adapted from the input samples.

$A_{1}$ is formed by $R=WZ^{i}$, which results in an m×n matrix. Then, a softmax operation is performed in column *j*, j=1,2,…,n of R, resulting in a maximum location, say, $j_{m}$ in that row *i*, and its value $r_{j_{m}}$. Then, this value $r_{j_{m}}$ is assigned to the *j*-th diagonal element $A_{1}$.

The AGCA module may be described as in Equation (16), which may be more conveniently expressed as follows:

(19) $$y=F_{AGCA}\left(x\right)$$

where x and y are, respectively, the input and output of the AGCA module and $F_{AGCA}\left(\cdot \right)$ represents the complex mapping described in this paragraph.

Since these weights formed the set of unknown weights that need to be trained, these weights are updated in each epoch through a backprop error.

### 3.4. The Overall Structure of the DB-MDA

As observed in Figure 3, this can be subdivided into two parts: The (VSS-SE) + (BA-AGCA) module and the NA module.

The (VSS-SE) + (BA-AGCA) module may be described by the following equations:

The pre-conditioning unit

The pre-conditioning unit may be described by the following equations:

(20) $$\begin{array}{ccc} z_{1} & = & F_{VSS//SE}\left(u_{1}\right) \end{array}$$

(21) $$\begin{array}{ccc} z_{2} & = & F_{VSS//SE}\left(u_{2}\right) \end{array}$$

where $u_{1}$ and $u_{2}$ are the two incoming inputs and $F_{VSS//SE}\left(\cdot \right)$ describes the function of the parallel configuration of the VSS and SE units. $z_{1}$, and $z_{2}$ are, respectively, the output of the pre-conditioning unit. Here we have not explicitly written the action of the Conv unit in each path for clarity of how the signal flows.

BA-AGCA unit

The signal flow may be described as follows:

(22) $$\begin{array}{ccc} z_{3} & = & F_{BA}\left(z_{1}\right) \end{array}$$

(23) $$\begin{array}{ccc} z_{4} & = & F_{BA}\left(z_{2}\right) \end{array}$$

where $F_{BA}\left(in\right)$ represents the BA acting on the input in.

Then, these two signals $z_{3}$$z_{4}$ are first expanded back to the same dimension as before the average operation, added elementally, and then divided into two signals $u_{3}$ and $u_{4}$.

Then, these two signals pass through the AGCA, and this is described as follows:

(24) $$\begin{array}{ccc} z_{5} & = & F_{AGCA}\left(u_{3}\right) \end{array}$$

(25) $$\begin{array}{ccc} z_{6} & = & F_{AGCA}\left(u_{4}\right) \end{array}$$

where $F_{AGCA}\left(in\right)$ represents the action of AGCA on the input in.

Then, the output of this preconditioning BA+AGCA unit is obtained as follows:

(26) $$z_{7}=u_{3}⊗z_{5}⊗u_{4}⊗z_{6}$$

where ⊗ stands for elementwise multiplication.

The NA residual network may be represented as follows:

(27) $$y=F_{NA}\left(z_{7}\right)+z_{7}$$

where y represents the output of this DB-MDA module.

Novelty of the DB-MDA module

Compared with the FER-NCAMamba model [30], which uses the same YOLOX framework for the SDAC task, the novelty of our model lies in the feature extractor used in the neck network. For FER-NCAMamba, it uses the same pre-conditioning unit: the parallel connection of the VSS module and the SE module followed by a feature extractor; let us denote it as $F_{NCAMamba}$, while in our model we use a weighted sum of the VSS output and SE output. Moreover, our feature extractor $F_{DB-MDA}$ is very different to that of $F_{NCA}$.

For $F_{NCAMamba}$, it uses Coordinate Attention (CA) and NA according to the following concept: The incoming signal is divided into two paths, called conveniently the X and Y coordinates of the incoming signal. Then, each of these would go through an NA before finally combining the output and passing through yet another NA. Conceptually, it uses the NA to extract local features along the X and Y directions, respectively. Then, these local features in the X direction and Y direction are fused and then pass through another NA, which will extract further local features of the combined signals.

In contrast, $F_{DB-MDA}$ passes the dual incoming signals independently through a BA, which extracts the global relationship among the samples in a batch. Then, these global features are fused together before being split into two separate signals. These two separate paths individually passes through an AGCA, which assigns a weight to each element in the incoming stream; these weights are adjusted dynamically according to the incoming samples. Then, all four paths are combined using elementwise multiplication before passing to a residual network containing the NA as the residual block. The residual network extracts local features in the spatial dimension.

To be specific, the differences lie in the following:

- Our DB-MDA model uses BA, while this is absent from that of FER-NCAMamba.
- DB-MDA does not use coordinate attention, while FER-NCAMamba uses it.
- DB-MDA deploys AGCA to extract channel weights dyanmically, one for each element of the input channels, while FER-NCAMamba uses channel attention as the final module before the output stage.
- DB-MDA uses NA in a residual network while FER-NCAMamba uses NA in a feedforward fashion together with coordinate attention.

As observed in Section 4.8, the performances in the testing dataset of RAF-DB differ: The balance between detection and classification is better for DB-MDA than for FER-NCAMamba.

## 4. Experiments

In this section, we will present the two datasets, RAF-DB and SFEW, with un-aligned images used in the experiments (see Section 4.1). Details on how some new images were obtained so as to evaluate the generalization capability of the trained models are all presented in Section 4.1. We will provide details on the experimental setup (see Section 4.2). We will then introduce the evaluation metrics used to assess the quality of the results (see Section 4.3). Moreover, we conducted an ablation study to investigate the relative importance of the key components of the attention module (see Section 4.4). Next, we provide a visualization analysis of the model’s predictions on the two datasets (see Section 4.5). This is followed by a presentation of the generalization capability investigations on the new images obtained (see Section 4.7). We compare the performance of our model with those of other state-of-the-art methods (see Section 4.6). Moreover, in Section 4.8, the interesting question of the differences in capability between the two top performing models, FER-NCAMamba and DB-MDA, is answered through applying the trained model to the testing dataset of RAF-DB to consider its misclassification errors and its number of undetected instances of expression in each category of expressions. Section 4.9 indicates some limitations of the proposed DB-MDA model.

### 4.1. Datasets

#### 4.1.1. RAF-DB and SFEW Datasets

In our research, we primarily used two datasets, RAF-DB (Real-world Affective Faces Database) [21] and SFEW (Static Facial Expressions in the Wild) [37] (both contained un-aligned images), for training and evaluating our proposed model. The un-aligned images in these two datasets will facilitate our study on the Simultaneous Detection and Classification (SDAC) of images that are closer to the real-life applications of FER, where it is not known *a priori* where a face is, or if there is only one face in the given image, how far the face(s) are from the camera, what the orientation of the face might be, or if part of the face is self-occluded; all these non-ideal realities are smoothed away if one first carries out a detection process and then normalizes the detected face to be located at the center of the image, and if necessary rotate it suitably. By insisting on SDAC, one is forced to consider the extraction of features in such a way that they will facilitate the detection of the occurrence of a face in the image, and then to classify the facial expression of the captured face accordingly.

Moreover, some images are collected to investigate the generalization capabilities of both trained models. Details of this will be presented in sequel.

The details of the RAF-DB and SFEW datasets are given in Table 1.

RAF-DB

RAF-DB [21] is a widely used dataset that contains a variety of real-life facial expressions, including emotions such as anger, disgust, fear, happiness, neutral, sadness, and surprise. The dataset includes 15,339 facial images, each labeled with its corresponding emotional category. The data in RAF-DB are sourced from the internet and real-world environments, making them highly authentic and diverse. In our study, we used the un-aligned version of RAF-DB, meaning that these images were not subjected to standard alignment processing, more accurately reflecting the facial expression variations seen in natural settings.

SFEW

The SFEW dataset [37] focuses on static snapshots of dynamic facial expressions, consisting of 1251 images selected from movies and video clips, displaying emotions such as anger, disgust, fear, happiness, neutral, sadness, and surprise. Similar to the RAF-DB dataset, the un-aligned version of the SFEW dataset was used, which has not undergone explicit alignment. These un-aligned SFEW images preserve the original facial features and expression variations from movies and videos, enabling the model to better handle the complexities encountered in real-world applications.

#### 4.1.2. Additional Images to Evaluate the Generalization Capabilities of Both Trained Models on Unseen Data

Some new images were obtained to evaluate the curiosity question: How would a trained model, say, using the training dataset of RAF-DB, perform on images that are collected under different environment, using different sensors to those used in the training data. Moreover, we like these images to be captured in real-life situations, but not with an intention to evaluate the limits of the trained model. So, the subjects were asked to stand some distances from the camera, not necessarily posing for the expression consciously, but subtly, among everyday scenes, e.g., in a computer laboratory with many other objects cluttered in the background, some with a banner with written English phrases of exhortation. There is only ever one person in the image. These environments are designed to capture simple facial expressions under everyday life scenarios. The question then becomes: How well would a trained model perform under such conditions?

Details of Camera Used

We used a camera with 2 million pixels. Its core is a 1/2.8—inch Sony CMOS sensor. Each pixel is precisely 2.9 μm by 2.9 μm. The sensor size is 5568 μm by 3132 μm. With a capture speed of about 16.67 milliseconds per frame, this camera can record dynamic scenes quickly. In low light conditions, the signal-to-noise ratio is greater than 69 dB, and in normal conditions it is greater than 42 dB. This ensures clear and high-quality images in various lighting conditions.

Details of the images captured

We used the camera and shot photos of nine subjects, capturing only two facial expressions: “Happy” and “Neutral”. These two facial expressions were chosen because they are the easiest to recognize from the experiments conducted (please see details in Section 4.6), ending up with a collection of 36 photos in total, i.e., 4 photos each, 2 for “Happy” and 2 for “Neutral”, respectively.

These images were used to test models trained on two datasets, namely SFEW and RAF_DB, respectively. We fed these images into both models for recognition and performed a detailed analysis of the results (see Section 4.7).

We will refer to this dataset as “Real Life” test dataset.

### 4.2. Implementation Details

The experiments in this study were conducted on a computer equipped with an NVIDIA A40 GPU, using the PyTorch (version 2.1.1) deep learning framework. The initial learning rate was set to $1\times 10^{-3}$, with the minimum learning rate set to $1\times 10^{-5}$. A cosine annealing learning rate decay strategy was employed to dynamically adjust the learning rate during training, helping to improve model convergence in the later stages. The entire training process lasted for 300 epochs, during which the model continually optimized the objective function to improve performance. For training on the SFEW dataset, the batch size was set to 50, while for the RAF-DB dataset it was set to 100. The reason for this configuration is that, when applying attention mechanisms along the batch dimension, different batch sizes yield varying effects for datasets of different sizes.

To enable the model to extract robust features and accelerate the training process, the backbone network was initialized with pre-trained weights on the COCO dataset [64]. Input images were standardized to 320×320×3 according to the network’s input requirements. Additionally, several simple data augmentation techniques were employed during training to enhance the model’s robustness, including random cropping, rotation, and flipping.

The hyperparameters used in our model are shown in Table 2.

Explanation of some of the terms used in Table 2

In the training process, there are a number of parameters that are used to control the learning. “Freezing” means the update in the weights would be frozen. So, in the batch attention, the freeze batch size is 16 and the unfreeze batch size for RAF-DB dataset would be 100, while it is 50 for the SFEW dataset. The freeze epoch and unfreeze epoch concern the update of the parameters in the backbone network. Freeze epoch means that the number of epochs for which the weights in the backbone network, starting with Init Epoch, will not be updated, while unfreeze epoch means the number of epochs for which the weights will be updated. In our case, we set Init epoch to 0 and freeze epochs to 0, i.e., the entire epoch of 300; the weights in the backbone network will be updated.

In post processing, we use an objectness confidence threshold of 0.5.

NMS stands for Non-Maximum Suppression. This is a method used in object detection to remove extra bounding boxes that are detected around the same object. When an object is detected multiple times with different bounding boxes, NMS keeps the best one and removes the rest. Here in the post processing of the outputs of the heads, we used a threshold of 0.3 so as to eliminate multiple bounding boxes of the same object.

These parameters were obtained through trial and error using some validation data from the training dataset. In some cases, a small grid search is required to find the optimal value; for example, the value for momentum. This term controls how much memory is retained from the previous iteration for the current iteration.

### 4.3. Evaluation Metrics

In this study, our experiment is based on the YOLO architecture [39] for multi-class object detection and classification tasks; the detection capability of the model is primarily characterized by the Average Precision (AP) as the evaluation metric. AP is used to measure the detection accuracy of the model at different thresholds, calculated by integrating the area under the precision–recall curve for each category. The specific calculation process is as follows:

- Precision and Recall Calculation:
  (28) $$Precision=\frac{TP}{TP+FP}$$
  (29) $$Recall=\frac{TP}{TP+FN}$$
  where TP represents the number of true positives, FP represents the number of false positives, and FN represents the number of false negatives.
  It is possible to combine the precision and recall into an F1 score:
  (30) $$F1=\frac{Precision\times Recall}{Precision+Recall}$$
- Average Precision (AP) Calculation:
  Average precision is the mean of precision values at different recall levels, calculated by integrating the precision–recall curve:
  (31) $$AP=\int_{0}^{1}P\left(r\right)dr$$
  where P(r) denotes the precision at recall *r*.
  Likewise, it is possible to compute the average recall and the average F1 accordingly.
- Mean Average Precision (mAP) Calculation:
  The mean of the AP values for all *N* categories is calculated as follows:
  (32) $$mAP=\frac{1}{N}\sum_{i=1}^{N}AP_{i}$$
  where N is the total number of categories and $AP_{i}$ is the average precision for the i-th category.
  The mAP reflects the model’s detection performance across all categories and provides an evaluation of the overall detection capabilities of the model.

For the evaluation on the Real Life dataset, due to its small number of samples, we used in addition the number of correctly classified and misclassified samples; for details, please see Section 4.7.

### 4.4. Ablation Experiments

We conducted one major ablation experiment: Evaluation of the relative effectiveness of BA, AGCA, NA, and VSS-SE of our proposed DB-MDA module within the YOLOX architecture.

Our work focuses on the feature extraction capabilities of the DB-MDA module within the YOLOX framework.

In our DB-MDA module, BA and AGCA are crucial components, so we conducted the following ablation experiments:

(i) the full module with all components in place (this corresponds to the first row in Table 3 and subsequent rows in the table followed the same convention).
(ii) removing BA by itself and replaced by a direct connection,
(iii) removing AGCA by itself, with the BA in place, and replaced the AGCA by a direct connection,
(iv) removing both BA and AGCA, and replaced by a direct connection,
(v) replace the dual branch BA, AGCA modules, and the NA module by a direct connection, with only the VSS-SE module in place,
(vi) removing the dual branch BA AGCA modules, the NA module, and the VSS-SE module, and replacing with a direct connection; in other words, there is no VSS-SE module, nor any of the attention components.

From these experiments, we can draw the following observations:

- BA, AGCA, and NA play a crucial role in enhancing the model’s performance. According to the experimental results shown in Table 3, when AGCA is used alone, the mAP decreases from 83.59% to 83.11%; when BA is used alone, the mAP decreases from 83.59% to 83.39%. However, when both BA and AGCA are removed simultaneously, mAP drops to 83.27%. When BA, AGCA, and NA are removed, mAP drops to 83.00%. Therefore, it can be inferred that BA, AGCA, and NA all contribute to the model’s performance to some extent. However, it should be noted that the performance when using both AGCA and NA is worse than when using NA alone. On the other hand, the performance when using BA, AGCA, and NA together is significantly better than when using only BA and NA.
  Based on these observations, we hypothesize that AGCA’s effectiveness in extracting image features depends on the presence of BA. In other words, BA influences the feature extraction results of AGCA. Given the fact that AGCA is downstream from the BA in the dual branch BA-AGCA architecture, this hypothesis is a reasonable one.
- Both modules (VSS-SE and attention) contribute to improving model performance, with the attention module having the more significant impact. When the attention module is removed, the mAP drops from 83.59% to 83.00%, demonstrating its effectiveness. The VSS module also enhances the model’s performance. When the VSS-SE module is removed alongside the attention module, the mAP further decreases from 83.00% to 82.38%.

### 4.5. Visualization Analysis

**Figure 6:**
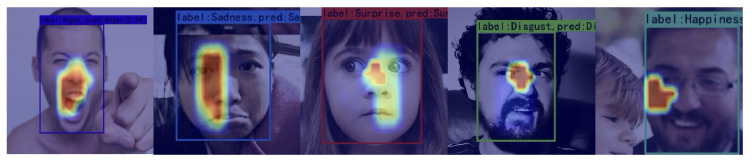
Grad-CAM visualization results of correctly classified samples on RAF-DB dataset. The rectangular region shown indicates the detected region for which an object would occur (from the detection part of the head network.

**Figure 7:**
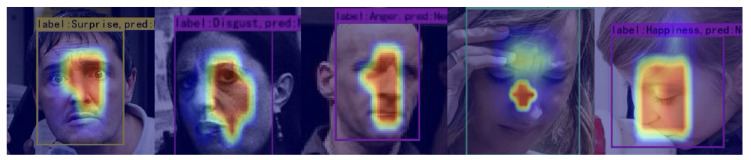
Grad-CAM Visualization Results of Incorrectly Classified Samples on RAF-DB Dataset.

**Figure 8:**
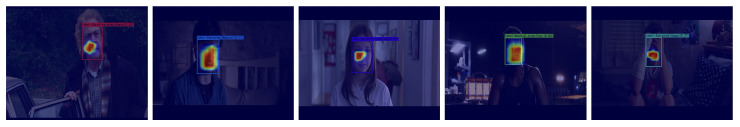
Grad-CAM visualization results of correctly classified samples on SFEW dataset.

**Figure 9:**
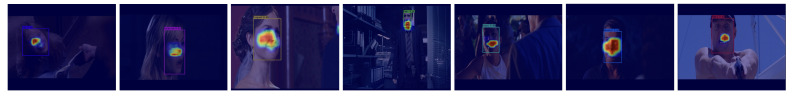
Grad-CAM visualization results of incorrectly classified samples on SFEW dataset.

To further analyze the model’s ability to learn facial expression characteristics, we used the Grad-CAM method (Gradient-Way Activation Mapping) [65] to visualize the prediction capabilities of the model. Figure 6 and Figure 7 respectively show some examples of correctly classified facial expressions and incorrectly classified RAF-DB dataset images, while their counterpart for the SFEW dataset are shown in Figure 8 and Figure 9, respectively. Grad-CAM uses class-specific gradient information to generate a heatmap that indicates the regions of the input image the model focuses on, helping to reveal its decision-making process.

As shown in Figure 6 for the RAF-DB dataset and Figure 8 and Figure 9 for the SFEW dataset, this is the Grad-CAM heatmap for correctly classified data. The heatmap generated by Grad-CAM is overlaid on the original image, with red areas highlighting the regions of the image that the model focuses on the most. From the visualization results, we observe that our model primarily focuses on the corners of the eyes, nose, and corners of the mouth on the human face, identifying expressions based on the curvature of the eye and mouth corners as well as the degree of mouth opening. This is particularly interesting for the SFEW dataset, as this dataset contains samples for which the face is not centered in the image, and its distance from the camera could be rather different. It is observed in Figure 8 the region in which the face is detected to occur is correctly found; moreover, the model correctly focus on the regions around the eye (see, e.g., (ii) in Figure 8), or around the mouth (see, e.g., (v) in Figure 8) despite its relatively small region when compared with the size of the image. The focus on such minute details on the face may be attributed mainly to the NA in the DB-MDA module. The ability to locate the face among a clutter of other objects, like those in the SFEW dataset, could be attributed to the dual BA-AGCA branches, as they seek to discriminate the information coming into both branches of which among the global features would be the possibility of a face, rather than other objects. Unfortunately, it is not possible to pin-point the contribution of each component in the DB-MDA module without a comprehensive analysis of each correctly classified face, and incorrectly classified ones using a brunt tool like Grad-CAM. Even though Grad-CAM is rather brunt, nevertheless it gives some impression on which areas on the detected object the model is focusing on to make a prediction of the possible facial expression class that the person is making.

However, in some cases, such as images with self-occlusions (see, e.g., face (ii) in Figure 7; (i) in Figure 9) or lighting variations, the Grad-CAM heatmap shows that the model’s attention may shift to irrelevant regions (see, e.g., in face (ii) in Figure 7, where Grad-CAM focuses on the “cheek”, which is largely irrelevant in classifying a particular facial expression). It is rather more difficult to discern from the Grad-CAM heatmap shown in Figure 7 why DB-MDA made an error in classifying the facial expression, when the face is reasonably well posed, see, e.g., face (iii) in Figure 7, and that Grad-CAM indicates a region, around the mouth, the tip of the nose, and the right eye, to varying degree of intensity, that the resulting classification is incorrect, compared with the ground truth information. This may be due to the imprecise manner in which a human is asked to classify the expression on a face, even by an expert following, say, the FACTS scheme [10], as the human expert is not obliged to give a detailed account of the reasons based on the subtle cues displayed by the facial muscles which led to the suggested classification, so using Grad-CAM or any other analysis tools, we are asking such tools to discover what might be the underlying reasoning that leads to a particular classification. This could be accomplished if there are a large number of examples, but not with the number of wrongly classified samples in the RAF-DB dataset. It could also be accomplished if the image information is augmented by physiological signal measurements, like from wearables.

The situation observed in images (ii) and (iii) of Figure 9 is interesting. For image (ii), while the DB-MDA model is able to detect the face, even though among a clutter of other objects, e.g., books, it focussed on the mouth and chin region of the person, and, probably because the chin is not prominent in any primary facial expression classification, it made a wrong classification of the facial expression. Image (iii) in Figure 9 is also very interesting; here there are two faces, one is well occluded, being not facing the camera, but facing the other person. The detection part of the proposed model correctly detected two occurrences of faces but could not distinguish which face is the main target in this image. It then focused on the region around the mouth and chin of the main targeted face but failed to correctly identify its facial expression. It may be that the context cues introduced by the other face interfered with those in the targeted face. Such possible interference is not explicitly considered in our model and yet this could play a role in detecting the targetted face in the presence of multiple faces in an image. It may be impossible to consider in a static situation, like in our case, and only possible in a dynamic situation, when the faces moved relatively to one another, for the algorithm to discern that there are multiple faces in the given short time segment.

It is rather more difficult to discern why image (v) in Figure 9 is misclassified. The face is well posed and the lighting condition appears to be ideal. But Grad-CAM seems to indicate that the model is focussed on the wrong region, even though it is around the tip of the nose and the upper lip. One could surmise that this might be an example to illustrate the difficulty of FER. A number of facial regions together compose a facial expression, but the exact composition is at best guesswork, even though FACTS [10] provides an extensive user manual to assist in correctly identifying the facial expression. This is more an art than exact science. Therefore, it is unsurprising to find situations when it is difficult to explain what the algorithm is doing.

### 4.6. Comparison with State-of-the-Art Methods

In this section, we compare our DB-MDA model with other state-of-the-art methods. In other to make it more comprehensive, we have included comparisons with other methods in the non-YOLO family, which applied directly only a particular classification technique to the datasets and did not depend on the detection of the face occurrence in the image. The results of such comparison are shown in Table 4. Table 5 shows the comparison of the Average Precision (AP), expressed in % of each category of facial expressions, between our proposed DB-MDA model and its nearest rival, as concluded from Table 4.

From Table 4 and Table 5, we can draw the following observations:

- It is noteworthy that, on both the SFEW and RAF-DB datasets, our method, DB-MDA, outperforms all other State-of-the-Art (SOTA) methods in terms of mAP. Specifically, on the RAF-DB dataset, our method achieves an mAP of 83.59%, while its closest competitor, FER-NCAMamba, achieves an mAP of 83.30%. On the SFEW dataset, our method attains an mAP of 69.43%, compared to FER-NCAMamba’s 68.66%. It is important to note that we used the un-aligned versions of the datasets, and therefore our results should not be compared with those obtained by methods that perform alignment on the samples before classification. In the latter case, the significance of detection would be greatly diminished. However, in such scenarios, the accuracy results may not be robust, as they are based on aligned information rather than on un-aligned samples.
  The superior performance of our model is attributed to our meticulously designed multi-dimensional attention mechanism. As ablation studies (see Section 4.4) have confirmed, each dimensional attention component within our multi-dimensional attention mechanism, regardless of its nature, contributes to the performance enhancement of the model—some significantly, others subtly—yet each is an indispensable force propelling our model forward.
- It is interesting to note that, in Table 4, almost all models find that “Happy” facial expression is the category that scores the best AP on both datasets. This may indicate that the expression “Happy”, because of the way it is expressed by most people by their facial muscles, is most obvious.
- When observing the performance of our model across different expression labels, we note that its efficacy varies with the size of the dataset. This is illustrated in Table 5.
  On the RAF-DB dataset, our model is better in recognizing images labeled as “Sad” and “Fear”, while its performance on other categories lags behind those of FER-NCAMamba, even though, in most cases, they are only behind in the first decimal place. This is why, on average, our model outperforms that of FER-NCAMamba, because in those two expressions, “Sad” and “Fear”, it has sufficient “head room” to provide for the overall lead. We hypothesize that, for the RAF-DB dataset, our model primarily focuses on the mouth region of the face, as the characteristics of Sad and Fear expressions are most pronounced in this area. This conjecture has been substantiated through Grad-CAM visualizations in Figure 6 and Figure 7.
  Similarly, our model achieves better results in identifying “Anger”, “Disgust”, “Happy”, and “Surprise” expressions on the SFEW dataset than those of the FER-NCAMamba, where the defining features of these emotions are predominantly located around the eyes, nose, and brow regions, as shown in Figure 8. Consequently, our model pays relatively more attention to these areas when processing SFEW images, a focus that has also been confirmed by Grad-CAM visualizations.
  These analyses shed some light on the capabilities of our model. However, these observations need to be treated with caution; there are only two datasets, and there are two methods: Ours and FER-NCAMamba. To draw more conclusive observations, one would be advised to consider more datasets, and more models rather than just two, even though these two are the ones that obtained the best and second best performances. It would be advisable to consider more models, and if possible more datasets, before making sweeping statements concerning the relationship between models and the size of datasets, even though intuitively this seems plausible. In dealing with such a challenging problem like FER from un-aligned images, where there could be labelling errors and subtle muscle movements on a face captured far from the camera, it pays to be cautious, as there could be “demons lurking around” to defy logic or common sense based on limited exposure to the data.

Table 5 raises another interesting question: Would the two models, DB-MDA and FER-NCAMamba, behave sufficiently different from one another? This question arises because of the perceptually small differences in their design: Both FER-NCAMamba and DB-MDA consist of two parts: the pre-conditioning part and feature extraction part. They have an almost identical pre-conditioning part, a parallel connection of two paths: VSS and SE. The difference is that, in DB-MDA, the relative importance between the contribution of the VSS component and that of the SE component would be learned rather than assumed to be the same as that in FER-NCAMamba. While the feature extraction part are different, they both made use of NA. Admittedly, in the case of FER-NCAMamba, it contains another attention mechanism, coordinate attention, while in the case of DB-MDA it involves other attention mechanisms: Batch Attention (BA) and a version of channel attention, Adaptive Graph Channel Attention (AGCA). The question then one may ask is: Would such seemingly small differences in this feature extraction module make much differences to their respective performances on the same dataset? This question is answered in Section 4.8.

### 4.7. Evaluation of the Generalizabilities of the Trained Models on the Real Life Dataset

Table 6 shows the results of applying trained models on the Real Life dataset.

From Table 6, it is observed that both the RAF-DB trained model and the SFEW trained model have poor performances in real-world environments.

Table 7 shows a breakdown of the details of recognition of both trained models. The term “correctly classified” means that the classification of the image matches that of the ground truth; “failed to recognize” means that faces are detected but the emotion scores—the multiplication of the objectness score of the bounding box detected and the classification score—is below a threshold of 0.5; and “misclassified” means that the predicted class does not match that of the ground truth.

From Table 6 and Table 7, the following observations may be made:

- In terms of accuracy, a model trained on the RAF-DB training dataset appears to be less accurate than the one trained on SFEW. Table 7 shows that the number of correctly classified images differs by only 1 (16 in the case of SFEW and 15 in the case of RAF-DB). This appears to contradict the intuition that RAF-DB has a larger number of training samples compared with that of SFEW and should provide a better recognition accuracy than that of SFEW.
  However, a deeper consideration of the nature between RAF-DB and SFEW and Table 7 could explain this anomaly. While the RAF-DB dataset contains un-aligned faces, it does not have the large variety of backgrounds like those occurring in the SFEW dataset. Moreover, the RAF-DB dataset contains fewer number of faces that are at a distance from the camera, while SFEW contains faces shot at considerable distance from the camera. This may explain some of the statistics as reported in Table 7. In Table 7, it is observed that the SFEW trained model makes nine instances of misclassification, while the RAF-DB trained model makes only two instances of mis-classification. This implies that RAF-DB trained model is better at correctly classifying the expression than that of a SFEW trained model. Moreover, for a SFEW trained model, it makes 11 instances of failed to recognize, while the RAF-DB trained model makes 19 instances of failed to recognize. This shows that the the RAF-DB trained model is less adept at recognizing faces shot at considerable distances from the camera, while the SFEW trained model is better adept for such occasions, as it has many more examples of such a situation occurring in its training dataset.
- Admittedly, here we have only 36 samples, and so any observations would need to be treated with caution. These can only be preliminary observations. But judging from the results of this experiment, a state-of-the-art model, like our DB-MDA model, is unlikely to be ready for deployment in real-life situations, even for relatively “lightweight’ applications like screening.
- This experiment brings to the foreground the importance of a large amount of training data with a large variety of backgrounds, and images shot with a variety of distances from the camera. However, collecting large-scale real-life data and having them labelled is expensive, and one would need a convincing use case before the expenses required could be justified. But then without convincing results from a model, it is hard to convince some institutions to invest vast sums of money without a likely good outcome in sight. This is the kind of “Catch22” situation in which researchers are caught. A good way forward would be to continuously improve on the models and slowly expand the types of data that could be used in evaluating the models to see how such models could be used on data that is more and more like real-life situations. Hopefully with enough evidence on “toy” datasets, with gradual relaxation in the direction of real-life situations, the area would be ready for commercial exploration.

**Figure 10:**
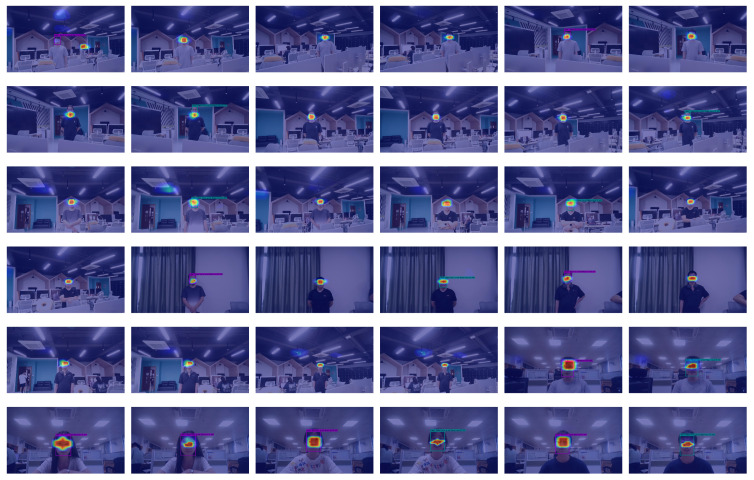
Visualization results of the RAF-DB trained model on the Real Life dataset.

**Figure 11:**
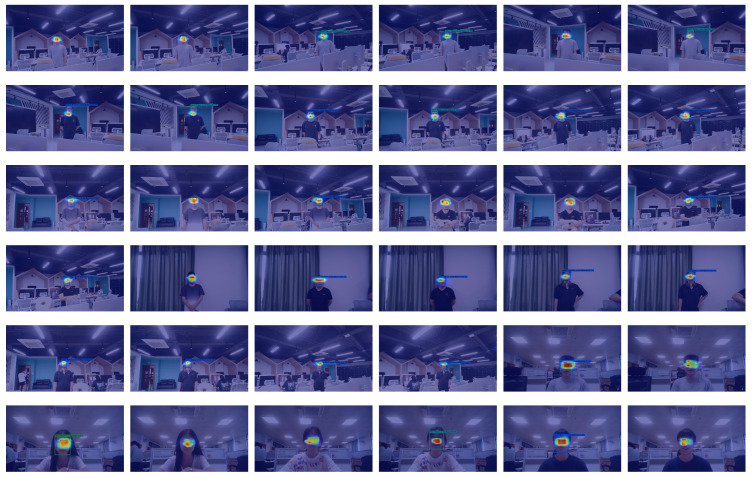
Visualization results of the SFEW trained model on the Real Life dataset.

Figure 10 and Figure 11, respectively, show the Grad-CAM visualization of all images in the Real Life dataset by the RAF-DB trained model and the SFEW trained model.

Due to the bruntness of the Grad-CAM tool, the following observations can only be taken as preliminary ones.

- It is observed that, despite the cluttered background in most images, both trained models appear to be able to find the bounding boxes that delimit the human faces in the image.
- While the face is correctly located, the region of the heatmap overlaid on the detected face cannot be discerned. They are often not focused on the regions in which the facial expression most likely would manifest, e.g., the corner of the mouth, the corner of the eye. Instead, sometimes it focuses on the chin, sometimes it is across the forehead, and sometimes, it is a “smudge” around the nose and mouth regions.
  This may point in the direction that the input image resolution used, 320×320, might not be high enough to reveal the fine details of such regions when the face is at a considerable distance from the camera. Moreover, it is observed that, where no bounding box is found (when the emotion score falls below a threshold of 0.5), the heatmap could fall on the general area for recognition.
  This may also point to the fact that the feature extractor designed is not sensitive enough to the fine features that are displayed in these regions. Admittedly, currently we only generate three scaled signals from the input image. It may be useful to experiment with a FPN with more scales than three. This may delineate if the issue arises due to the lack of high-resolution inputs.

### 4.8. Discussions on the Differences in the Capability of FER-NCAMamba and DB-MDA Models

We decided to run an experiment on the same dataset to understand the capabilities of our DB-MDA model when compared with those of FER-NCAMamba. In particular, we wish to consider a version of the confusion matrices of applying either trained model to the testing dataset of RAF-DB. The version of confusion matrix used is the standard confusion matrix except with the diagonal elements removed to highlight the misclassification errors. Moreover, it includes an extra column to denote the number of undetected instances of the expression for each category of facial expressions considered. We denote this as a modified confusion table. This will allow us to compare the detection and classification capabilities of a trained model. The resulting confusion tables are shown in Table 8 and Table 9, respectively.

The following observations may be made from these two tables, Table 8 and Table 9:

- It is observed that the FER-NCA model and the DB-MDA model have a different balance between the undetected and the classified errors. The DB-MDA model appears to have struck a better balance between classification accuracies and un-detected errors. The DB-MDA model achieves a total number of misclassifications of 309 when compared with that of the FER-NCA model of 337, while it had 163 undetected faces and FER-NCA had 159 undetected faces.
- It appears that, for the FER-NCA model, the categories in which it made most mistakes in classification when compared with those of DB-MDA would be “Happiness” and “Neutral”, and in every other categories, except in the category of “Disgust”, it made slightly less errors than that of the DB-MDA model.
- These results show conclusively that these two models are doing different things on the RAF-DB dataset: FER-NCA makes a lesser balance on the classification and detection capacities than those of DB-MDA.
- The question of why DB-MDA achieves a better balance between classification and detection would require extensive investigations of the qualities of the features extracted by either model, especially in relation to the global features for detection and the fine features used in classification, with respect to their designs. This task would be a good topic for future work.

### 4.9. Limitations of Our DB-MDA Model

Our DB-MDA model, even though it achieves results that are comparable to other SOTA methods, nevertheless has some limitations.

- Investigations reported in Section 4.7 reveals an uncomfortable truth about most deep learning methods: They works well on “toy” datasets, where the training dataset and the testing dataset are obtained in the same environment. However, if the trained model is applied to samples that are collected using different environments, then the performance degrades, sometimes dramatically. This is typified in the experiment we conducted in Section 4.7.
  A way forward would be to explore few shot learning (see, e.g., [70]), in which new samples collected from different environments could be incorporated through this methodology. A sideline of this research, which may be more immediate, would be to explore more sophisticated data augmentation schemes, like those used in [70]. This would increase the diversity of images, and thus would make the recognition more robust.
  In the longer term, there is a need to collect more data under a variety of backgrounds, a variety of distances between the subject and the camera, more than one subject in some of the images, and under various lighting conditions, and label them accordingly. The availability of large-scale FER datasets would facilitate the development of improved models.
- As shown clearly in Section 4.8, it strikes a better balance than the FER-NCA model in apportioning the conflicting demands of global features for detection purposes and fine features for good classification of facial expressions; there are still 10% misclassfied samples and 5% undetected faces in the testing dataset of RAF-DB. Depending on the application, e.g., screening, such margins of errors could be tolerable. However such error margins might not sit comfortably if this is used for diagnosis purposes. But then, if the idea of FER from static images is used together with other physiological measurements, an ensemble type of method could be used to combine the individual physiological measurements and FER and such an error margin could be tolerable. A suitably designed ensemble method would improve the accuracy in determining the emotions of the person.
- Currently, the loss function used to train the model consists of three components: the cross entropy loss, the IoU (Intersection over Union) loss for determining the location of the bounding boxes and the ground truth information, and the objectness confidence indicating the probability of object presence. These loss functions could suitably be augmented by other measures. For example, within AGCA, there is no control on the magnitude of the elements of the matrix $A_{2}$. While every effort has been taken to condition these elements to behave well, like initializing them with very small values, and through small learning rates and high momentum terms, the very idea that some of these elements could grow without limits is uncomfortable, even though, on the two datasets used to evaluate the model, RAF-DB and SFEW, no observable growth is discerned in all experiments. It is possible to include in the loss function a regularization term, on the size of the elements of matrix $A_{2}$, and to ensure its diagonal elements will be close to 0. This would force the elements to stay within some bound and the diagonal elements to be close to 0.
- Among the three attention mechanisms used, both the operation in the channel dimension and in the spatial dimension are currently the most sophisticated ones available; only the batch attention is still quite crude, as it uses the self-attention mechanism in the batch dimension. It is perceivable that one could use a version of neighborhood attention along the batch dimension.
- The attention mechanisms used is one way of extracting features. There are other developments in the area of feature extraction, e.g., in emphasizing the role of discriminative regression projection in feature extractions (see, e.g., [71]). Incorporating features extracted using other ideas than attention mechanism might improve the types of feature extracted for SDAC tasks. This would be a fruitful area of further research.

## 5. Conclusions

This paper addresses a major issue in the simultaneous detection and classification of facial expressions: a good balance between two competing demands: the need to have good global features to facilitate detection and the need to have good fine features to facilitate good classification. To that end, we propose a novel architecture, which replaces feature extraction in the “neck” network of the YOLOX framework. This novel architecture consists of two parts: a pre-conditioning part, followed by a dual branch multi-dimensional attention (DB-MDA) part. The pre-conditioning part consists of two parallel paths: a Visual State Space (VSS) path and a Squeeze and Excite (SE) path; the relative contribution of these two paths are learned through adjustable weights. The DB-MDA module consists of an innovative deployment of three attention mechanisms: batch attention for processing in the batch dimension; adaptive graph channel attention to devise an individual weight for each element in the input tensor (BS×C×H×W), where *BS* is the batch size, *C* is the number of input channels, and H×W represents the spatial dimension; and neighborhood attention to process the spatial dimension.

This novel feature extraction module embedded is in the YOLOX framework and is applied to two non-aligned FER datasets, RAF-DB and SFEW, and it was found that it has competitive performance when compared with other state-of-the-art methods. Moreover, an experiment was conducted to investigate the generalizability capability of the trained model on samples that are not collected in the same environment as those in RAF-DB and SFEW. It was found that the performance degraded significantly.

Through experiments on the testing data of the RAF-DB dataset by two trained models, the FER-NCAMamba model and our own DB-MDA model, it is found that the DB-MDA model achieves a better balance between the need to have good global features for detection purposes and the need to have good fine features for good classification of the facial expressions when compared with the FER-NCAMamba model. Moreover, it is demonstrated through these experiments that these two models have quite different behaviors.

There are limitations in our DB-MDA model, chief among them being the lack of an explicit balancing between the features for good detection and features that are good for classifications. Moreover, the lack of an explicit means to ensure that the elements of the AGCA module are contained within some bound means that it would be uncomfortable to consider deployment of this DB-MDA model to practical uses, except maybe in screening. These would form good topics for future research. Moreover, soon, one could begin to consider the more challenging problem of simultaneous detection, tracking, and classification of facial expressions, which is closer to the requirements of the practical deployment of FER to daily living.

## Figures and Tables

**Table 1:** Detailed size of the experimental dataset.

Datasets	Train Size	Test Size	Classes
RAF-DB [21]	12,271	3068	7
SFEW [37]	1125	126	7

**Table 2:** Hyperparameters used in our model.

Category	Parameter	Value
Training	Input Shape	(320, 320, 3)
	Freeze Batch Size	16
	Unfreeze Batch Size(RAF-DB)	100
	Unfreeze Batch Size(SFEW)	50
	Init Epoch	0
	Freeze Epoch	0
	Unfreeze Epoch	300
	Init Learning Rate	0.001
	Min Learning Rate	$1\times 10^{-5}$
	Optimizer	Adam
	Momentum	0.937
	Learning Rate Decay Type	cosine
Post-processing	Confidence Threshold	0.5
	NMS Threshold	0.3

**Table 3:** Performance comparison of different feature extraction modules on the RAF-DB dataset. In the table, “✓” indicates the presence of the module, while “→” indicates a direct connection.

Methods	Components				mAP (%)	Avg_F1	Avg_Recall (%)	Avg_Precision (%)
	VSS-SE	BA	AGCA	NA				
RAF-DB	✓	✓	✓	✓	83.59	0.78	76.48	80.00
	✓	→	✓	✓	82.80	0.78	75.67	79.84
	✓	✓	→	✓	83.39	0.78	76.11	79.83
	✓	→	→	✓	83.27	0.78	76.10	80.76
	✓	→	→	→	83.00	0.79	76.31	81.00
	→	→	→	→	82.38	0.77	76.01	78.31

**Table 4:** Performance comparison of different methods on RAF-DB and SFEW datasets. Figures shown in bold indicate the best performing in the group of competing methods.

Methods	Year	mAP(%)		Best AP(%)	
		SFEW	RAF-DB	SFEW	RAF-DB
SSD [66]	2015	59.22	77.89	91.20 (Happy)	95.72 (Happy)
RetinaNet [67]	2017	56.67	75.63	81.87 (Happy)	94.63 (Happy)
YOLOv3 [22]	2018	19.18	59.42	50.28 (Happy)	88.09 (Happy)
CenterNet [68]	2019	28.48	59.92	68.57 (Happy)	91.32 (Happy)
EfficientNet [69]	2019	15.87	71.45	29.68 (Happy)	93.75 (Happy)
YOLOv4 [23]	2020	12.31	42.23	29.58 (Anger)	87.61 (Happy)
YOLOv5 [24]	2020	13.63	50.15	25.52 (Neutral)	91.77 (Happy)
YOLOvX [25]	2021	64.02	78.40	90.81 (Happy)	96.82 (Happy)
YOLOv7 [26]	2022	52.02	68.17	74.34 (Happy)	92.01 (Happy)
YOLOv8 [27]	2023	51.94	72.09	87.50 (Happy)	93.33 (Happy)
YOLOv10 [28]	2024	64.81	79.58	88.61 (Happy)	94.18 (Happy)
FER-YOLO-Mamba [29]	2024	66.67	80.31	90.94 (Happy)	97.43 (Happy)
FER-NCAMamba [30]	2024	68.66	83.30	86.50 (Happy)	95.31 (Happy)
Ours	-	**69.43**	**83.59**	**91.26** (Happy)	**95.16** (Happy)

**Table 5:** AP (%) comparison between Ours and FER-NCAMamba across expressions. Figures shown in bold indicate the best performing one in the group.

Expression	SFEW		RAF-DB	
	Ours	FER-NCAMamba [30]	Ours	FER-NCAMamba [30]
Anger	**76.29**	75.75	84.27	**85.82**
Disgust	**79.22**	75.25	63.01	**63.24**
Fear	55.68	**61.56**	**69.11**	67.32
Happy	**91.26**	86.50	95.16	**95.31**
Neutral	46.41	**59.38**	**90.74**	90.92
Sad	**69.36**	66.03	**90.16**	88.99
Surprise	**67.78**	56.16	91.37	**91.46**
Average	**69.43**	68.66	**83.59**	83.30

**Table 6:** The performance of RAF-DB and SFEW trained models on the Real Life dataset in %. Details of recognition of both trained models on the Real Life test dataset are given in Table 7.

Model	RAF_DB	SFEW
Accuracy	41.67	44.45
mAP	45.45 ^1^	45.45 ^2^

^1^: For RAF-DB, the mAP of “Neutral” is 0.4545, the mAP of “Happy” is 0.4545, the average is 0.4545. ^2^: For the SFEW, the mAP of “Neutral” is 0.6364, the mAP of “Happy” is 0.2727, the average is 0.45455

**Table 7:** Detailed breakdown of the number of correctly classified samples, samples which were not recognized, or misclassified. Please see text on the explanation of the differences among these categories.

Dataset	Correctly Classified	Failed to Recognize	Misclassified	Total
RAF-DB	15	19	2	36
SFEW	16	11	9	36

**Table 8:** The modified confusion table of FER-NCAMamba model on the RAF-DB test set.

True Label	Predicted Class								Undetected
	Anger	Disgust	Fear	Happiness	Neutral	Sadness	Surprise	Total	
Anger	–	9	3	4	8	3	1	28	13
Disgust	4	–	0	15	22	12	1	54	22
Fear	1	0	–	4	3	9	8	25	7
Happiness	3	9	1	–	26	8	5	52	38
Neutral	0	8	0	18	–	46	11	83	38
Sadness	1	8	0	8	39	–	3	59	19
Surprise	3	2	3	7	15	6	–	36	22
Total	12	36	7	56	113	84	29	337	159

**Table 9:** The modifed confusion table of Ours on the RAF-DB test set.

True Label	Predicted Class								Undetected
	Anger	Disgust	Fear	Happiness	Neutral	Sadness	Surprise	Total	
Anger	–	6	2	5	9	1	0	23	16
Disgust	8	–	0	9	25	15	2	59	17
Fear	2	1	–	3	4	6	8	24	5
Happiness	4	6	0	–	24	5	4	43	42
Neutral	2	10	0	17	–	40	6	75	34
Sadness	4	7	2	7	34	–	0	54	24
Surprise	3	2	6	3	13	4	–	31	25
Total	23	32	10	44	109	71	20	309	163

## Data Availability

The original contributions presented in the study are included in the article, further inquiries can be directed to the corresponding author.
